# Lysophosphatidic Acid Enhanced the Angiogenic Capability of Human Chondrocytes by Regulating Gi/NF-kB-Dependent Angiogenic Factor Expression

**DOI:** 10.1371/journal.pone.0095180

**Published:** 2014-05-30

**Authors:** Yi-Wen Chuang, Wen-Ming Chang, Kai-Hua Chen, Chang-Zern Hong, Pey-Jium Chang, Hung-Chih Hsu

**Affiliations:** 1 Department of Physical Medicine and Rehabilitation, Chia-Yi Chang Gung Memorial Hospital, Chia-Yi, Taiwan; 2 Department of Nursing, Chang-Gung University of Science and Technology, Chia-Yi, Taiwan; 3 Department of Physical therapy, HungKuang University, Taichung, Taiwan; 4 Graduate Institute of Clinical Medical Sciences, College of Medicine, Chang Gung University, Taoyuan, Taiwan; University of Texas Southwestern Medical Center, United States of America

## Abstract

Lysophosphatidic acid (LPA) has been found to mediate myeloid differentiation, stimulate osteogenesis, alter cell proliferation and migration, and inhibit apoptosis in chondrocytes. The effect of LPA on the angiogenic capability of chondrocytes is not clear. This study aimed to investigate its effect on the angiogenic capability of human chondrocytes and the underlying mechanism of these effects. Human chondrocyte cell line, CHON-001, commercialized human chondrocytes (HC) derived from normal human articular cartilage, and human vascular endothelial cells (HUVECs) were used as cell models in this study. The angiogenic capability of chondrocytes was determined by capillary tube formation, monolayer permeability, cell migration, and cell proliferation. An angiogenesis protein array kit was used to evaluate the secretion of angiogenic factors in conditioned medium. Angiogenin, insulin-like growth factor-binding protein 1 (IGFBP-1), interleukin (IL)-8, monocyte chemoattractant protein-1 (MCP-1), matrix metalloproteinase (MMP)-9, and vascular endothelial growth factor (VEGF) mRNA and protein expressions were evaluated by Q-RT-PCR and EIA, respectively. LPA receptor (LPAR) expression was determined by RT-PCR. Signaling pathways were clarified using inhibitors, Western blot analysis, and reporter assays. The LPA treatment promoted the angiogenic capability of CHON-001 cells and HC, resulting in enhanced HUVEC capillary tube formation, monolayer permeability, migration, and cell growth. Angiogenin, IGFBP-1, IL-8, MCP-1, MMP-9, and VEGF mRNA and protein expressions were significantly enhanced in LPA-treated chondrocytes. LPA2, 3, 4 and 6 were expressed in CHON-001 and HC cells. Pretreatment with the Gi/o type G protein inhibitor, pertussis toxin (PTX), and the NF-kB inhibitor, PDTC, significantly inhibited LPA-induced angiogenin, IGFBP-1, IL-8, MCP-1, MMP-9, and VEGF expressions in chondrocytes. The PTX pretreatment also inhibited LPA-mediated NF-kB activation, suggesting the presence of active Gi/NF-kB signaling in CHON-001 and HC cells. The effect of LPA on the angiogenesis-inducing capacity of chondrocytes may be due to the increased angiogenesis factor expression via the Gi/NF-kB signaling pathway.

## Introduction

Lysophosphatidic acid (LPA) is a naturally occurring phospholipid, which can have either a cellular (e.g., cancer cells, fibroblasts, adipocytes, and platelets) or non-cellular (e.g., lipoprotein) origin [1]. LPA also has a variety of physiologic and pathologic functions [2]–[8], regulating cell survival, apoptosis, motility, shape, differentiation, gene transcription, malignant transformation, as well as other processes [9], [10]. LPA acts through the cell surface G protein–coupled receptors, LPA1, LPA2, LPA3, LPA4, LPA5 and LPA6, which mediate a wide range of human cellular responses [11].

The chondrocyte cell line, CHON-001, is widely used in chondrocyte-related studies; it was derived from the long bones of an 18-week female fetus. The primary cells were infected by a defective retrovirus containing the *human telomerase reverse transcriptase* (*hTERT)* gene under G418 selection [12]. Nowadays, primary culture of chondrocytes derived from normal human articular cartilage is also commercially available for researches. Several studies have revealed that LPA mediates myeloid differentiation within the human bone marrow microenvironment [13] and stimulates osteogenesis [14], cell proliferation [15], and migration [16] and inhibits apoptosis [17] in chondrocytes.

The bone structure formed and enlarged from the proliferation and differentiation of mesenchymal cells condensates into chondrocytes [18]–[20]. At this time, cells in the center stop proliferating and become hypertrophic chondrocytes, which generate the surrounding matrix. Bone collar formation ensues after collagen-expressing cells attract blood vessels and induce adjacent perichondrial cell differentiation into osteoblasts. During vascular invasion, osteoblasts appear in the primary spongiosa and begin to synthesize new bone. Thus, angiogenesis is critical for bone formation. Several studies revealed that partial loss of vascular endothelial growth factor (VEGF) proteins in mice impairs skeletal angiogenesis and delays chondrocyte hypertrophy, bone formation, and cartilage calcification [21], [22]. However, the effect of LPA on the angiogenic effects of chondrocytes is not clear. In this study, we evaluated the effects of LPA on the angiogenesis-stimulating capacity of chondrocytes by examining the expression pattern of angiogenesis-related factors. In addition, the molecular mechanism of LPA-induced angiogenic factor expression was determined in the chondrocyte cell line, CHON-001 and human chondrocytes (HC), derived from normal human articular cartilage.

## Materials and Methods

### Cell culture

Human chondrocyte cell line, CHON-001, and human umbilical vein endothelial cells (HUVECs) were obtained from the American Type Culture Collection (Rockville, MD, USA). Human chondrocytes (HC) were purchased from Cell Applications (San Diego, CA, USA).CHON-001 cell line was maintained in DMEM (Life Technologies, NY, USA), 0.1 mg/ml G-418 supplemented with 10% (vol/vol) fetal bovine serum (FBS). Chondrocytes (HC?) (2×10^6^ cells/10-cm plate) were maintained in chondrocyte growth medium (Cell Applications; San Diego, CA, USA) for 24 h. HC from passages five to eight were used in this study. HUVECs were maintained inM199 medium supplemented with 20% FBS, endothelial cell growth supplement (Intracel, Rockville, MD, USA), heparin, L-glutamine, penicillin, and streptomycin. HUVECs from five or more different donors were pooled together to prevent possiblegenetic variations caused by sampling. HUVECs were used at no more than five passages.All cells were cultured in a humidified atmosphere of 95% air and 5% CO_2_ at 37°C.

### LPA and chemical inhibitors

Oleoyl-LPA, fatty acid-free bovine serum albumin, PTX, and PDTC were purchased from Sigma (St Louis, MO). LPA was dissolved in 1X PBS containing 1% fatty-acid-free bovine serum albumin.

### Preparation of condition medium (CM)

Cells were washed with PBS twice and cultured in 5 mL of serum-free DMEM for 24 hours both before and after the 1 hour treatment with LPA or vehicle. CM was then collected and clarified by centrifugation (4°C, 10000 rpm, 5 min) from cell debris. A solution of 25 mM HEPES buffer (pH 7.4), 1 mg/ml leupeptin, 1 mM phenylmethylsulfonyl fluoride, 1 mM EDTA, 0.02% NaN3, and 0.1% BSA (Sigma) was next added to CM. The CM was lastly frozen and stored at −70°C before being used.

### HUVECs tube formation assay

HUVECs (2×10^4^ cells/well in 96 well plate) were plated onto a thin coating of Matrigel (0.24 mg/cm^2^) with CM. HUVECs are a primary (non-transformed) endothelial cells, they form tubes within 6 h.(Nature Protocols 5, 628–635;2010).After 6 hours, three random fields of wells were digitally photographed.

### HUVECs proliferation test

Cells were plated onto six-well cell culture plates at 1×10^5^ cells/well in 2 ml of culture medium with CM. After 72 h of treatment at 37°C, cells were harvested by suspension in 0.025% trypsin in 0.02% EDTA solution. Cell counts were performed in triplicate using a hemocytometer. Trypan blue exclusion assay was used to identify viable cells.

### HUVECs monolayer permeability assay

HUVECs were cultured in Transwell chambers (0.4 ml pore polycarbonate filters; Costar, Cambridge, Miss., USA). After reaching cell confluency, the medium was replaced with CM (0.3 ml in the upper chamber and 1 ml in the lower chamber). Horseradish peroxidase molecules (type VI-A, 44 kDa; Sigma-Aldrich) at a concentration of 0.126 µM was added to the upper compartment. After incubation for 1 h, the medium in the lower compartment was assayed for enzymatic activity using a photometric guaiacol substrate assay (Sigma-Aldrich).

### HUVECs migration assay

A total of 2×10^4^ HUVECs in 200 µl of culture medium was added to the upper chamber of the 24-well Millicell inserts (8 µm pore; Millipore Corporate, Bedford, MA, USA) with 500 µl of culture medium added to the lower chamber. After cells had attached to the insert (approximately 6 hours of incubation), the culture medium was changed to serum-free culture medium in the upper chamber and to the conditioned medium (CM) in the lower chamber. The numbers of migrated HUVECs were lastly counted at 6 h after cell attachment to inserts.

### Protein array analysis

Proteome Profiler Human Angiogenesis Antibody Array (Catalog # ARY007, R&D Systems) was used for the angiogenesis related factors detection according to the manufacturer's instruction. Briefly, CM was first mixed with the detection antibody cocktail at room temperature for 1 h prior to being added to the array membrane. The membrane was then incubated overnight at 2–8°C on a shaker. After washing, horseradish peroxidase–conjugated streptavidin was next added to the membrane followed by 30 minutes incubation at room temperature on a shaker. After washing, X-ray film and a chemiluminescence imaging system were used to detect and quantify the array signals.

### Enzyme immunoassay

The protein levels of angiogenin, IGFBP-1, IL-8, MCP-1, MMP-9, and VEGF in the cell culture supernatant were determined using EIA kits from R & D according to the manufacturer's instructions. Each measurement was performed in triplicate.

### Real-time quantitative RT-PCR

The angiogenin, IGFBP-1, IL-8, MCP-1, MMP-9, and VEGF cDNA were analyzed using a fluorescein quantitative real-time PCR detection system (LightCycler DNA Master SYBR Green I; Roche Molecular Biochemicals, Indianapolis, IN). The primer pairs were showed in Table 1. For Amplification was followed by melting curve analysis to verify the correctness of the amplicon. A negative control without cDNA was run with every PCR to assess the specificity of the reaction. Analysis of data was performed using LightCycler software (Roche Diagnostics Ltd., Burgess Hill, UK). PCR efficiency was determined by analyzing a dilution series of cDNA (external standard curve). The amount of angiogenin, IGFBP-1, IL-8, MCP-1, MMP-9 and VEGF mRNA were normalized by that of glyceraldehyde-3-phosphate dehydrogenase (GAPDH) mRNA and were presented in arbitrary units, with 1 U corresponding to the value in cells treated with a vehicle control.

**Table 1:** The primer pairs of angiogenin, IGFBP-1, IL-8, MCP-1, MMP-9, VEGF and GAPDH for real-time quantitative RT-PCR.

gene	sequence (5′- 3′)
**Angiogenin**	**F:CCTGTGTTGGAAGAGATGGT R:CCTGTGGTTTGGCATCATAG**
**IGFBP-1**	**F:GCACGGAGATAACTGAGGAG R:TCCATGGATGTCTCACACTG**
**IL-8**	**F:TTTCTGCAGCTCTCTGTGAGG R:CTGCTGTTGTTGTTGCTTCTC**
**MCP-1**	**F:ATAGCAGCCACCTTCATTCC R:TTCCCCAAGTCTCTGTATCT**
**MMP-9**	**F:GGCGCTCATGTACCCTATGT R:TCAAAGACCGAGTCCAGCTT**
**VEGF**	**F:TCTTCAAGCCATCCTGTGT R:CTTTCTTTGGTCTGCATTC**
**GAPDH**	**F:GGGAAGGTGAAGGTCGG R:TGGACTCCACGACGTACTCAG**

### LPA specific receptors determination by RT-PCR

RT–PCR was used to verify LPA specific receptor expression. Total RNA was isolated from CHON-001 cell line using RNAzol B reagent according to the manufacturer's instructions. cDNA was prepared from 2 µg of total RNA with random hexamer primers according to the cDNA synthesis ImProm-II protocol (Promega). The specific oligonucleotide primer pairs and the expected sizes of the PCR products are showed in Table 2. The PCR products were electrophoresed on a 1.8% agarose gel at 100 V and visualized using ethidium bromide.

**Table 2:** The primer pairs of LPA specific receptors for RT-PCR.

gene	sequence (5′- 3′)	expected sizes
**LPA1**	**F:CAAAATGAGGCCTTACGACGCCA R:TCCCATTCTGAAGTGCTGCGTTC**	**621 bp**
**LPA2**	**F:CCTACCTCTTCCTCATGTTC R:ATGAGCAGGAAGACAAGCA**	**361 bp**
**LPA3**	**F:CTGATGTTTAACACAGGCCC R:GACGTTGGTTTTCCTCTTGA**	**402 bp**
**LPA4**	**F:GTTTCCGCATGAAAATGA GAA R:TGGAAAACAAAGAGGCTGAAA**	**342 bp**
**LPA5**	**F:CTAACCTCG TCATCTTCCTGCT R:GAAGGAAGACAGAGAGTGGGAGT**	**377 bp**
**LPA6**	**F:TTACTTCACAACACGGAATTGG R:TATGTTTTCCATGTGGCTTCTG**	**315 bp**
**beta-actin**	**F:CTTCTACAATGAGCTGCGTG R:TCATGAGGTAGTCAGTCAGG**	**305 bp**

### Western blotting

The nuclear and cytoplasmic proteins of CHON-001 cells were extracted by Nuclear and Cytoplasmic Extraction Kit (Thermo Scientific. IL, USA). The protein concentration was measured using a Bio-Rad protein assay. 10-µg protein samples were separated by 10% of sodium dodecyl sulfate polyacrylamide gel electrophoresis (SDS-PAGE) and then transferred onto the polyvinylidene difluoride (PVD) membrane. The membranes were blocked with 5% fat-free milk for non-phosphorylated form of protein or with 5% BSA for phosphorylated form of protein for 30 minutes. The membranes were then immunoblotted with various primary antibodies; anti-p50 and anti-p65 antibodies (Santa Cruz Biotechnology), for 1 hour at room temperature. The bound antibodies on the membranes were detected using peroxidase-coupled secondary antibodies for 30 minutes. Signals were detected by X-ray film with an enhanced chemiluminescent detection system.

### Promoter construction and reporter assays

The transfection of the nuclear factor (NF)-κB binding site-driven luciferase plasmid (BD Bioscience, Palo Alto, CA) into chondrocytes was performed in six-well plates using the Transfast transfection reagent (Promega). At 24 h after transfection, cells were serum starved for 24 h and then treated with the indicated conditions. To control the transfection efficiency, cells were cotransfected with pSV-β-galactosidase. Data normalizations after all transient transfections were conducted using triplicate cultures.

### Statistics

In this study, each experiment was repeated at least three times. Data were presented as mean ± standard deviation (SD). Non-parametric tests were used to evaluate statistically significant differences between the LPA treatment group and the vehicle control group in specified tests. ANOVA was used when there were more than two experiment groups and Tukey–Kramer multiple comparison procedures were used for *post hoc* testing. Pearson's correlation was used to test the relationship between two continuous variables. A *p-*value of <0.05 was selected as the statistical significance cutoff.

## Results

### LPA treatment significantly enhanced the angiogenic capability of chondrocytes

**Figure 1:**
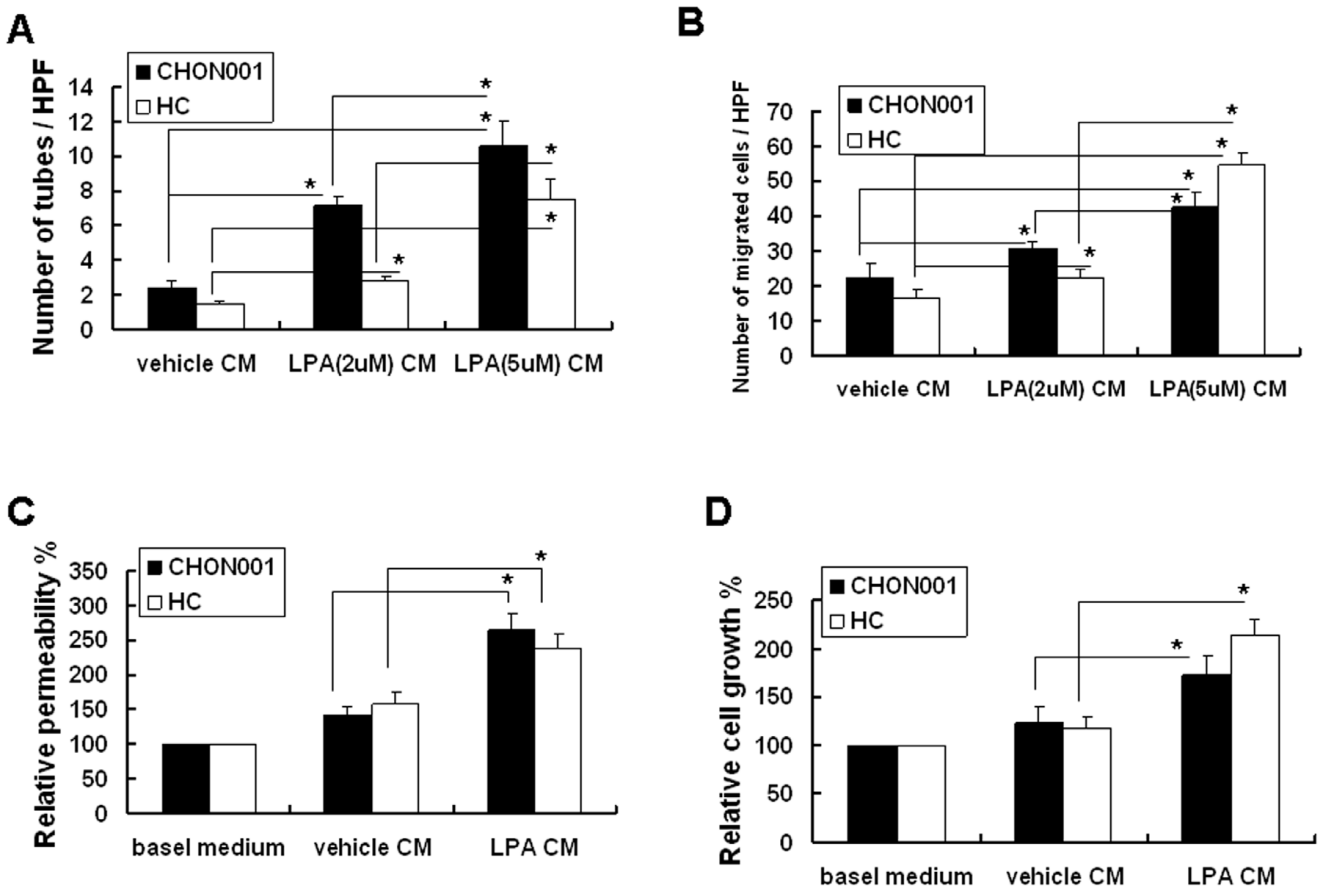
LPA treatment promotes the angiogenic capability of chondrocytes. Condition media (CM) from vehicle or LPA-treated cells were used for the following angiogenic function assays. (A) CM from vehicle or 2 uM or 5 uM LPA-treated cells was used for the HUVEC capillary tube formation assay. Data presented here were the quantitative results of the number of HUVEC tubes. (B) CM as described in (A) was used in the HUVEC migration assay. Data presented here were the quantitative results of the number of migrating HUVECs. (C) CM from vehicle or 5 uM LPA-treated cells was used in HUVEC monolayer permeability assay. Data are the relative permeability percentages resulting from the indicated conditions, in which the basal medium is defined as 100%. Comparison is between the indicated groups. (D) CM as described in (C) was used in HUVEC proliferation assay. Data were the relative cell number percentages obtained for the indicated conditions, in which basal medium was defined as 100%. n = 5 for each assay. ^*^ *p*<0.05.

To evaluate the effect of LPA on the angiogenesis in chondrocytes, we first treated CHON-001and HC with physiological levels of LPA (2 µM and 5 µM) for 1 h. After cell washing by PBS, fresh serum-free culture medium was added. After 24 h, the conditioned medium was collected. *In vitro* angiogenesis assays with HUVECs revealed that conditioned medium from 2 µM and 5 µM LPA-treated CHON-001 or HC significantly enhanced endothelial tube formation (Figure 1A) and endothelial migration (Figure 1B). Meanwhile, the statistical analysis between the 2 µM and 5 µM LPA treatment groups revealed that the effects of LPA on the angiogenesis in chondrocytes were in dose-response manner. Furthermore, the results of endothelial cell permeability (Figure 1C) and endothelial cell proliferation (Figure 1D) revealed that, relative to the vehicle-treated group, the conditioned medium from 5 µM LPA-treated CHON-001 chondrocytes or HC significantly enhanced the endothelial cell permeability and endothelial cell proliferation. Taken together, these data revealed the role of LPA on the enhancement of the angiogenic capacity of chondrocytes.

### LPA treatment significantly increased angiogenic factor expression by chondrocytes

**Figure 2:**
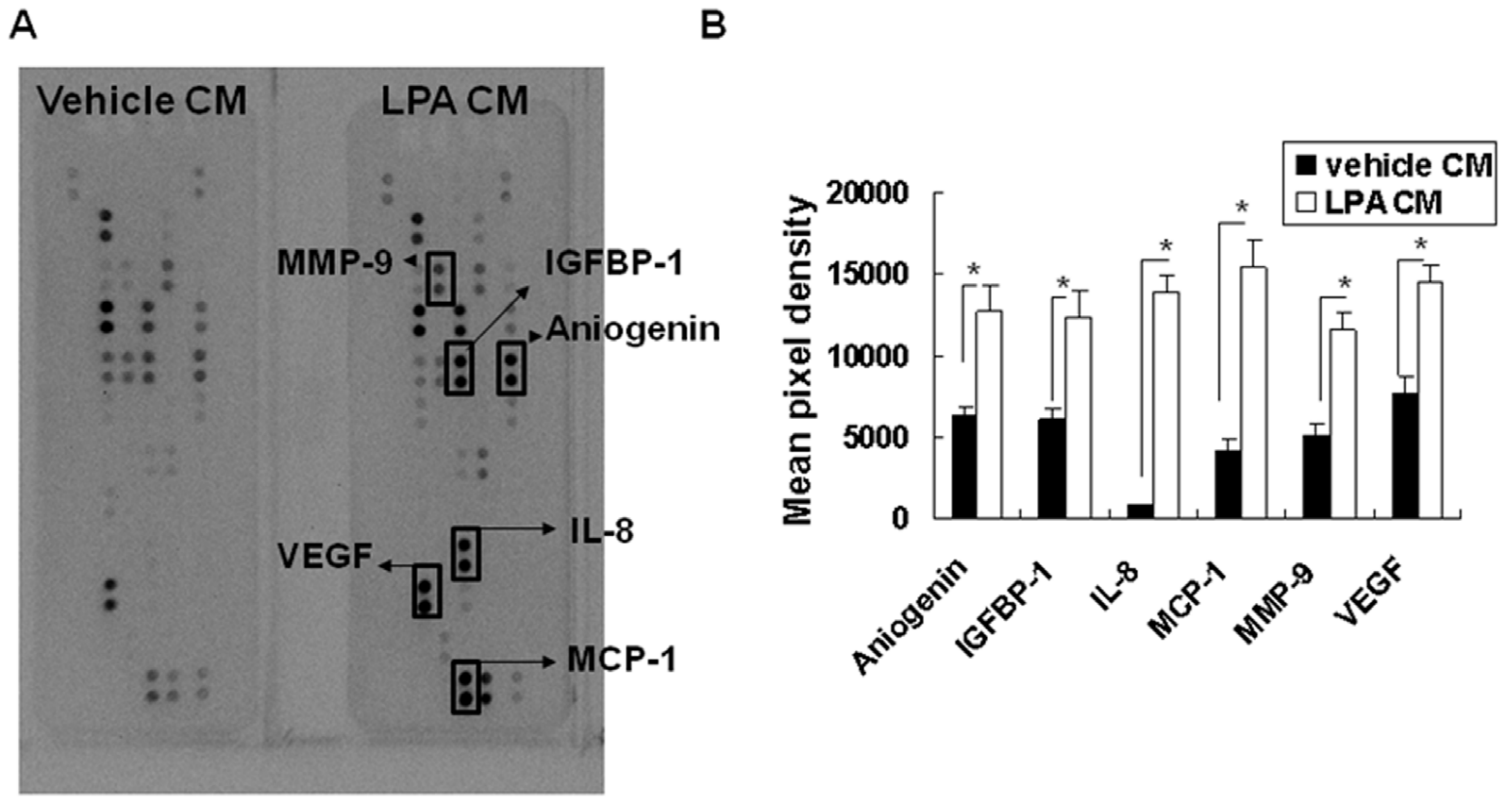
Angiogenesis factor protein secretion by LPA-treated cells. Conditioned media (CM) from vehicle- or LPA-treated cells were used for the parallel determination of the relative levels of human angiogenesis-related proteins. (A) Representative images of the angiogenesis protein array. The spots on the membrane incubated with LPA–CM with stronger signals than the vehicle-CM were labeled. The image shown was representative of three independent experiments. (B) Quantitative results of the angiogenesis protein array. Data represented the mean pixel density of the indicated proteins. n = 3; ^*^ *p*<0.05.

To clarify possible angiogenic factors within the conditioned medium from LPA-treated CHON-001, a protein antibody array was employed. As shown in Figure 2A, the LPA treatment enhanced the signals of angiogenin, insulin-like growth factor-binding protein 1 (IGFBP-1), interleukin (IL)-8, monocyte chemoattractant protein-1 (MCP-1), matrix metalloproteinase (MMP)-9, and VEGF. Histogram profiles for selected angiogenic factors were generated by quantifying the mean spot pixel densities from the array membrane using an image software (Figure 2B). The resultsrevealed that LPA significantly enhanced angiogenin, IGFBP-1, IL-8, MCP-1, MMP-9, and VEGF secretion by CHON-001.

### Effects of LPA on angiogenin, IGFBP-1, IL-8, MCP-1, MMP-9, and VEGF mRNA and protein expressions in chondrocytes

**Figure 3:**
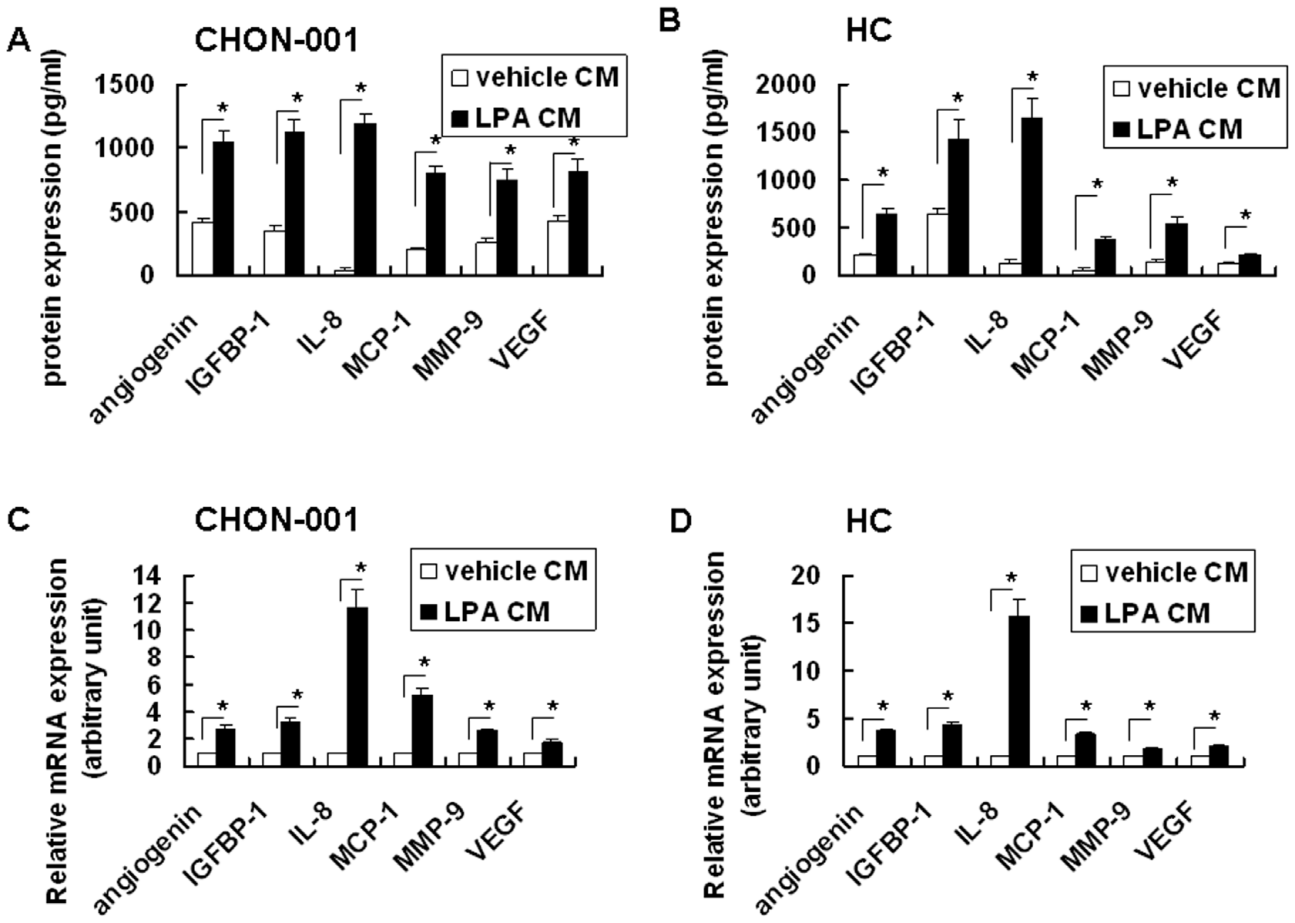
LPA significantly induced angiogenin, IGFBP-1, IL-8, MCP-1, MMP-9 and VEGF expression in CHON001and HC. Cells were treated with 5 µM LPA or vehicle (1% BSA). (A) After 24 h, the cell culture supernatant from CHON001 was used to detect angiogenin, IGFBP-1, IL-8, MCP-1, MMP-9 and VEGF proteins by EIA. Data were compared between indicated groups. (B) After 24 h, the cell culture supernatant from HC was assayed as described in (A). (C) CHON001chondrocytes were treated with 5 µM LPA or vehicle (1% BSA). After 6 h, mRNA levels of angiogenin, IGFBP-1, IL-8, MCP-1, MMP-9 and VEGF were determined by real-time quantitative RT-PCR and compared. (D) HC was treated with 5 µM LPA or vehicle (1% BSA). After 6 h, mRNA was assayed as described in (C). Data were compared between indicated groups. n = 3 for each assay. ^*^ *p*<0.05.

The levels of angiogenin, IGFBP-1, IL-8, MCP-1, MMP-9, and VEGF proteins in the conditioned media from both the vehicle- or LPA-treated CHON-001 or HC were next determined (Figure 3A and 3B, respectively). The results revealed that LPA significantly enhanced the levels of angiogenin, IGFBP-1, IL-8, MCP-1, MMP-9, and VEGF proteins in the cell culture supernatant of CHON-001 (Figure 3A) and HC (Figure 3B). Furthermore, QRT-PCR analysis revealed that LPA induced angiogenin, IGFBP-1, IL-8, MCP-1, MMP-9, and VEGF mRNA expressions in both CHON-001(Figure 3C) and HC (Figure 3D).

### LPA-regulated angiogenin, IGFBP-1, IL-8, MCP-1, MMP-9, and VEGF expressions in chondrocytes were mediated by Gi/NF-kB signaling

**Figure 4:**
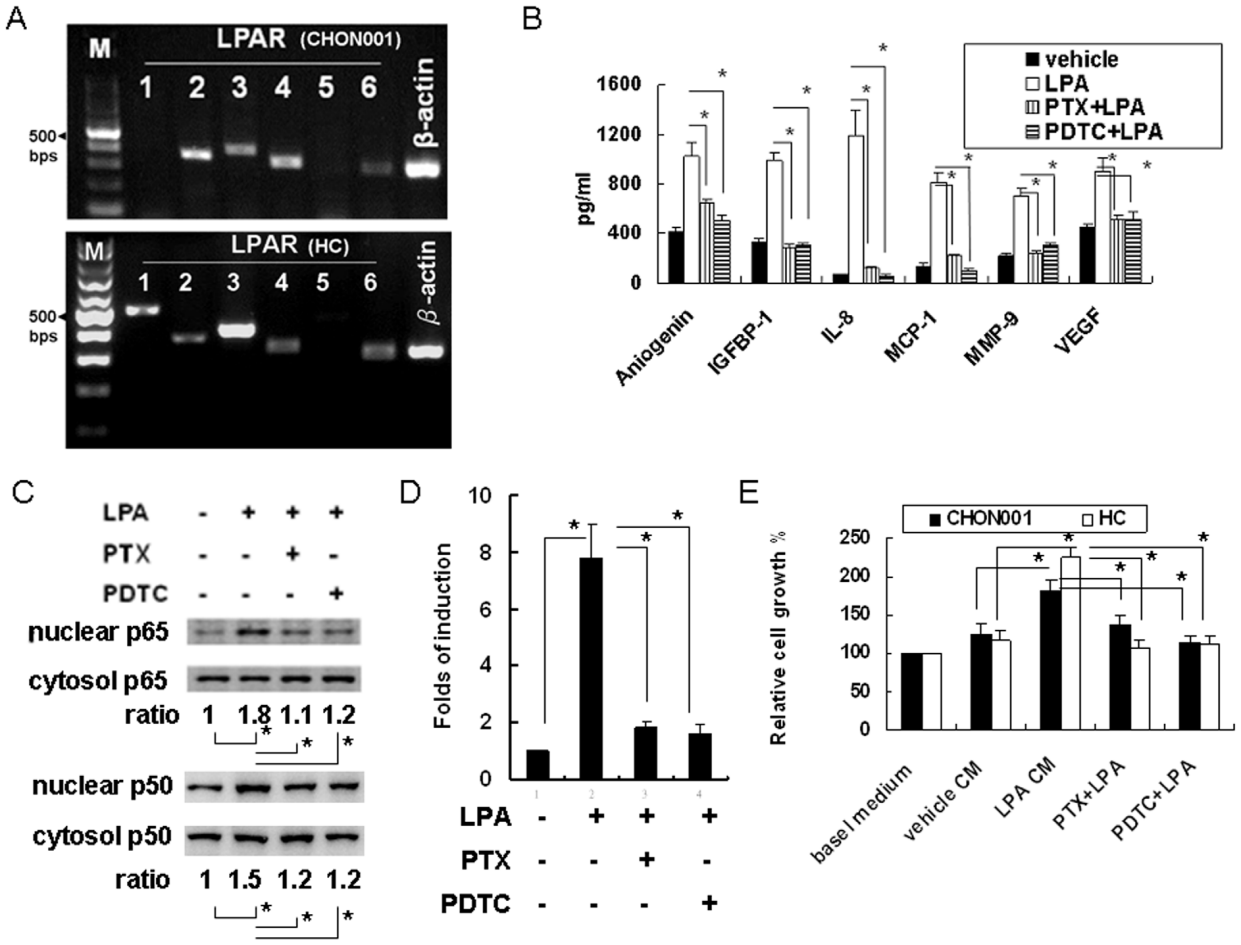
LPA-induced angiogenic factor expression was mediated through Gi/NF-κB signaling in chondrocytes. (A) Expression of LPA receptors in CHON001 chondrocytes and HC were detected by RT-PCR. The gel shown was representative of three independent experiments. (B) CHON001 chondrocytes were pretreated with PTX (100 ng/mL) or PDTC (100 nM) for 1 h prior to the LPA treatment. The protein levels of angiogenin, IGFBP-1, IL-8, MCP-1, MMP-9 and VEGF expressions were determined after 24 h by EIA. Data were compared with the vehicle-treated group. n = 5. (C) Cells were pretreated with PTX (100 ng/mL) or PDTC (100 nM) for 1 h prior to LPA treatment. The protein levels of NF-kB p50 and p65 in nuclear or cytosolic fractions were determined by Western blot analysis. The quantitative results are presented as the ratio of nuclear-to-cytosolic p50 or p65. Data were compared with the LPA-treated group. n = 3 (D) Cells were transfected with an NF-κB–driven luciferase reporter and then pretreated with indicated inhibitors for 1 h prior to LPA treatment. After 4 h, NF-κB promoter activities were determined. Data are compared between indicated groups. n = 5; ^*^ *p*<0.05. (E) CHON-001 cells and HC were pretreated with PTX (100 ng/mL) or PDTC (100 nM) for 1 h prior to 5 uM LPA treatment for CM collection as described in the materials and methods. CM was used for HUVEC proliferation assay. Data were the relative cell number percentages obtained for the indicated conditions, in which basal medium was defined as 100%. n = 5 for each assay. ^*^ *p*<0.05.

**Figure 5:**
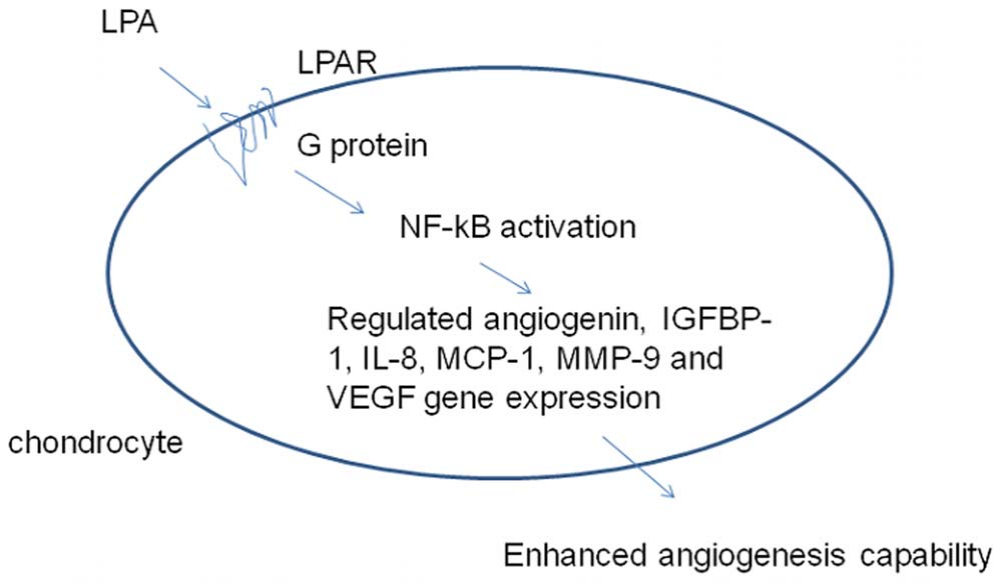
Schematic showing the mechanism by which LPA-induced signaling enhanced angiogenesis factor expressions in chondrocytes, resulting in enhanced angiogenic capacity.

The action of LPA is mediated through its receptor, LPAR. Therefore, we next determined the expression pattern of LPARs in CHON-001and HC by RT-PCR analysis. As shown in Figure 4A, LPA2, 3, 4, and 6 were expressed in CHON-001; LPA1, 2, 3, 4, and 6 were expressed in HC. Since LPAR might activate G protein-coupled receptors and subsequent downstream gene expression, CHON-001 cells were next treated with the Gi protein inhibitor, PTX, which could inactivate Gi/o type G proteins. PTX inhibited LPA-induced angiogenin, IGFBP-1, IL-8, MCP-1, MMP-9, and VEGF expressions in chondrocytes (Figure 4B). Meanwhile, we also identified critical transcription factors required for the angiogenic gene regulation by LPA in CHON-001. By analyzing the transcription factor binding sites within these genes, the NF-κB binding site was found to be presented in each of these genes. Therefore, we used the NF-κB inhibitor, PDTC, to evaluate its role in mediating angiogenic factor expression in CHON-001. PDTC significantly reduced LPA-mediated angiogenin, IGFBP-1, IL-8, MCP-1, MMP-9, and VEGF expressions in CHON-001 (Figure 4B). The canonical pathway of NF-κB activation is mediated by the p65 and p50 subunits, including nuclear translocation and DNA-binding activity. To verify the relationship between Gi and NF-κB signalings, cells were pretreated with PTX or PDTC prior to the LPA treatment. The LPA-induced NF-κB activation was determined by the detection of the p65 and p50 subunits subcellular distribution by Western blot analysis (Figure 4C). The result revealed that PTX as well as PDTC inhibited LPA-induced NF-κB p65 and p50 subunits nuclear translocation. The result also revealed the LPA-induced NF-κB activation with p50 and p65 heterodimers in articular chondrocytes. Furthermore, transfection of CHON-001with an NF-κB promoter reporter for 24 h and pretreated with PTX prior to the LPA treatment revealed that LPA significantly induced NF-κB reporter activity, which was significantly inhibited by PTX and PDTC (Figure 4D). Thus, LPA-induced NF-κB activation was mediated by upstream Gi signaling. The result revealed that the Gi/NF-κB signaling cascade plays a critical role in the LPA-induced angiogenesis-related protein expression. We further blocked the Gi/NF-kB signaling in CHON-001and HC both by PTX and PDTC pretreatments before the LPA stimulation and then collected the CM. The angiogenesis-inducing capacity of LPA was then verified by determining the cell proliferation of HUVEC. The result revealed that the blocking of the Gi/NF-kB signaling in CHON-001and HC by PTX or PDTC significantly inhibited the angiogenesis-inducing capacity of LPA (Figure 4E). Taken together, we conclude that the LPA mediated angiogenesis-inducing capacity of chondrocytes is mediated via the Gi/NF-kB signaling pathway (Figure 5).

## Discussion

Several studies have revealed the biological effects of LPA in chondrocytes. Panupinthu et al. have demonstrated that osteogenesis is suppressed in calvarial cell cultures from P2rx7-/- mice compared with wild-type mice. Functional P2X7 receptors are expressed by osteoblasts *in situ* and *in vitro*and P2X7 receptor signaling produces LPA and COX metabolites, which in turn stimulate osteogenesis [23]. The effects of LPA on chondrocyte-mediated angiogenesis have been verified in this study using 5 µM LPA, which is within the range of plasma LPA levels reported in healthy adults (<0.1–6.3 µM) [24].

The nutrients, oxygen, metabolic substrates, and circulating cells that help to support new bone formation all originate from the vasculature [25]. Thus, the regulation of angiogenesis is critical for bone formation. During angiogenesis, angiogenic growth factors diffuse into nearby tissues, binding to specific receptors located on the endothelial cells located around preexisting blood vessels and activateactivating them. Upon activation, endothelial cells proliferate and migrate to form a blood vessel. In this study, we used HUVECs as an endothelial cell model to determine the effects of LPA on chondrocyte-driven angiogenesis [26] by assessing endothelial permeability, migration, tube formation, and cell proliferation. Conditioned medium from the LPA-treated chondrocytes had an angiogenesis-enhancing effect.

In order to clarify possible angiogenic growth factors contained in the conditioned medium from chondrocytes, we used a commercialized human angiogenesis array to simultaneously detect the relative expression levels of 55 angiogenesis-related proteins. After the LPA stimulation, angiogenin, IGFBP-1, IL-8, MCP-1, MMP-9, and VEGF levels were significantly enhanced in the conditioned medium. Angiogenin enhanced neovascularization of experimentally injured menisci [27]. In addition, the family of IGF-binding proteins (IGFBP-1 to -6) is important in the regulation of IGF availability and bioactivity [28]. Although IL-8 is found to induce chondrocyte hypertrophy [29], it also induces endothelial permeability by transactivating the VEGF receptor-2 [30]. Enhanced expression of MCP-1 is observed in cultured human articular chondrocytes in response to oxidized low-density lipoprotein and may be involved in cartilage degeneration [31].Immunosuppression by MCP-1 has also been observed [32]. However, the role of MCP-1 during bone formation requires further investigation. Using MMP-9-/- transgenic mice, Ortega et al. report that the organization of hypertrophic cartilage extracellular matrix at the MMP-9-/- growth plate is altered, supporting a role for MMP-9 in hypertrophic cartilage remodeling. Furthermore, the inhibition of VEGF impairs osteoclast recruitment, which along with MMP-9 and VEGF, is necessary for normal endochondral bone formation [33].

LPA exhibits multiple biological activities via interaction with its specific receptor. The expression pattern of LPARs in human chondrocytes has not been previously clarified. In this study, we found that LPA2, 3, 4, and 6 were expressed in CHON-001 and LPA1, 2, 3, 4, and 6 were expressed in HC. Previous studies have revealed that LPA may enhance IL-8 expression through LPA1 in osteoblasts [34] and endothelial cells [35].LPA2 and 3 are critically involved in LPA-mediated IL-8 expression in cervical cancer cells [36] and in the LPA-mediated VEGF expression in ovarian cancer cells [37]. These references suggest that LPA1, 2, and 3 may play important roles in the LPA-mediated angiogenic capability of chondrocytes. Different signaling transduction mediators, including EGFR, PI3K/Akt, MAPK/p38, MAPK/ERK, PKC, JNK, NF-κB, and AP-1, are activated in LPA-treated cells. To identify a common signaling pathway responsible for the LPA-enhanced angiogenin, IGFBP-1, IL-8, MCP-1, MMP-9, and VEGF expressions, we determined the role of Gi signaling using PTX. The results reveal that Gi is critically involved in the regulated expression of these angiogenic proteins by LPA. Furthermore, promoter analysis indicates that NF-κB is a common transcription factor that regulates the expression of these genes [38]–[42]. The role of NF-κB in the LPA-enhanced angiogenin, IGFBP-1, IL-8, MCP-1, MMP-9, and VEGF expressions was verified using the NF-κB inhibitor, PDTC [43]. We have found that LPA-mediated NF-κB activation in chondrocytes is dependent on Gi signaling.

Thus, we conclude that LPA is a potent angiogenesis-promoting molecule in chondrocytes by inducing angiogenin, IGFBP-1, IL-8, MCP-1, MMP-9, and VEGF expressions via Gi/NF-kB signaling.
